# Staphylococcal lipoproteins and peptidoglycans synergize to drive skin abscess formation

**DOI:** 10.1128/mbio.00840-26

**Published:** 2026-05-15

**Authors:** Majd Mohammad, Zhicheng Hu, Julia M. Scheffler, Mulugeta Nega, Arif Luqman, Malgorzata Krzyzowska, Martina Sundqvist, Pradeep Kumar Kopparapu, Rille Pullerits, Abukar Ali, Minh-Thu Nguyen, Friedrich Götz, Tao Jin

**Affiliations:** 1 Department of Rheumatology and Inflammation Research, Institute of Medicine, The Sahlgrenska Academy, University of Gothenburg 214435, Gothenburg, Sweden; 2 Department of Microbiology and Immunology, The Affiliated Hospital of Guizhou Medical University 74720, Guiyang, China; 3 Department of Microbial Genetics, University of Tübingen 9188, Tübingen, Germany; 4 Biology Department, Institut Teknologi Sepuluh Nopember 561939 , Surabaya, Indonesia; 5 Department of Pharmaceutical Chemistry and Biomaterials, Faculty of Pharmacy, Medical University of Warsaw 417849, Sosnowiec, Poland; 6 Department of Clinical Immunology and Transfusion Medicine, Sahlgrenska University Hospital 56749, Gothenburg, Sweden; 7 Section of Medical and Geographical Infectiology, Institute of Medical Microbiology, University Hospital of Münster 235555, Münster, Germany; 8 Department of Rheumatology, Sahlgrenska University Hospital 56749, Gothenburg, Sweden; University of Colorado Anschutz Medical Campus School of Medicine, Aurora, Colorado, USA

**Keywords:** *Staphylococcus aureus*, lipoproteins, peptidoglycan, TLR2, skin infection, mouse, neutrophils

## Abstract

*Staphylococcus aureus* is a leading cause of skin and soft tissue infections (SSTIs), which can escalate into systemic disease. While innate immune responses play a critical role in bacterial clearance, the bacterial components themselves can exacerbate inflammation. Here, we demonstrate that *S. aureus* lipoproteins (Lpp) and polymeric peptidoglycan (PG) synergistically induce skin abscesses in mice, in a process that requires both the lipid moiety of Lpp and the intact polymeric structure of PG. This synergy is mediated by Toll-like receptor 2 (TLR2) and nucleotide-binding oligomerization domain-containing protein 2 (NOD2) and depends on infiltrating neutrophils and monocytes. Co-administration of Lpl1 and PG results in a 5-fold to 10-fold increase in macrophage inflammatory protein-2 (MIP-2) levels in the skin compared to either ligand alone, indicating a clear synergistic effect. Furthermore, we show that local alteration in coagulation and fibrinolysis contributes to the inflammatory response, as fibrinogen depletion significantly reduced lesion size. To extend these findings to a clinically relevant model, we employed an *S. aureus* double mutant that lacked both lipidation (Δ*lgt*) and peptidoglycan O-acetyltransferase (Δ*oatA*). This strain exhibited markedly attenuated virulence in a murine skin infection model. Importantly, this attenuation was fully reversed by neutrophil depletion, indicating that neutrophils are essential mediators of the host responses to these bacterial structures. Our findings reveal a cooperative mechanism through which *S. aureus* cell wall components drive skin lesion development, and we identify potential therapeutic targets for reducing the severity of SSTIs.

**IMPORTANCE:**

*Staphylococcus aureus* is a bacterium that often causes skin infections, including painful abscesses. We discovered that two components of *S. aureus*, lipoproteins on its surface and peptidoglycan in its cell wall, collaborate to drive the formation of skin abscesses. This combination triggers potent immune responses by activating the receptor Toll-like receptor 2 (TLR2) and nucleotide-binding oligomerization domain-containing protein 2 (NOD2) on host cells. As a consequence, high numbers of neutrophils and monocytes swarm the infection site. The resulting immune overreaction, together with activation of the coagulation system, produces intense inflammation. We confirmed the importance of these bacterial components using mutant *S. aureus* strains in a skin infection model. These mutants generated much smaller abscesses in our experiments. Our findings highlight a cooperative mechanism that exacerbates staphylococcal infections. Targeting this synergy could be a valuable strategy to reduce disease severity.

## INTRODUCTION

Skin and soft tissue infections (SSTIs) are among the most common types of infections, affecting a significant portion of the population at some point in life (1). *Staphylococcus aureus* is the predominant microorganism isolated from these infections, largely due to its ability to colonize the skin (1, 2). The clinical manifestations of *S. aureus* skin infections are highly variable, ranging from mild, superficial infections to severe, life-threatening conditions such as necrotizing fasciitis (1, 3). Importantly, SSTIs often serve as the initial site and entry point for more severe systemic infections. When left untreated or improperly managed, these infections may progress to more serious conditions, such as bacteremia and sepsis. In such cases, the infection spreads to other vital organs, including the brain, heart valves, and joints, leading to potentially fatal complications (4).

The innate immune system in skin tissues plays a crucial role in eliminating invading microbes using various factors, including antimicrobial peptides, the complement system, and reactive oxygen species, along with the granular proteins produced by infiltrating neutrophils. Interleukin-1 (IL-1)- and IL-17-mediated immune responses are particularly important in promoting the recruitment of neutrophils to sites of infection in the skin (3). As these immune defenses target and kill microbes, bacterial components are released and exposed to the immune system. Some of these components can induce proinflammatory reactions (5–7), potentially contributing to disease progression by acting synergistically.

Two components of *S. aureus* are of particular interest in this context. First, *S. aureus* lipoproteins (Lpp), which represent a major class of surface proteins, trigger immune activation via Toll-like receptor 2 (TLR2) and play a distinct role in disease pathogenesis, depending on the route of infection (8, 9). Second, peptidoglycan (PG), which is the most-abundant component of *S. aureus*, is recognized mainly by a receptor localized in the cytoplasm that lacks transmembrane domains, nucleotide-binding oligomerization domain-containing protein 2 (NOD2) (10). The stimulation by *S. aureus* PG of NOD2 in dendritic cells is enhanced when there is co-stimulation with Lpp *in vitro* (11), although this phenomenon has not been demonstrated in animal models. Due to their high stickiness and potent TLR2 agonist activity, Lpps are difficult to completely remove as contaminants (12). To minimize Lpp contamination, it is recommended to isolate *S. aureus* PG from a strain lacking lipidation (Δ*lgt*).

In the present study, we investigate the synergistic roles of Lpp and PG in inducing skin lesions *in vivo* by injecting these components subcutaneously (s.c.) into mouse skin. We identify the molecular structures of both bacterial components responsible for this effect. Furthermore, we explore the immunological mechanisms underlying this synergy by depleting various host immune cells and assessing the importance of the balance between coagulation and fibrinolysis through fibrinogen depletion in animal models. Finally, we use an *S. aureus* strain that lacks both lipidation and peptidoglycan O-acetyltransferase in a murine model of skin infections to better understand the roles of Lpp and PG in *S. aureus*-mediated skin infections.

## RESULTS

### Co-injection of *S. aureus* Lpp and PG induces a synergistic inflammatory reaction in mouse skin

**Fig 1:**
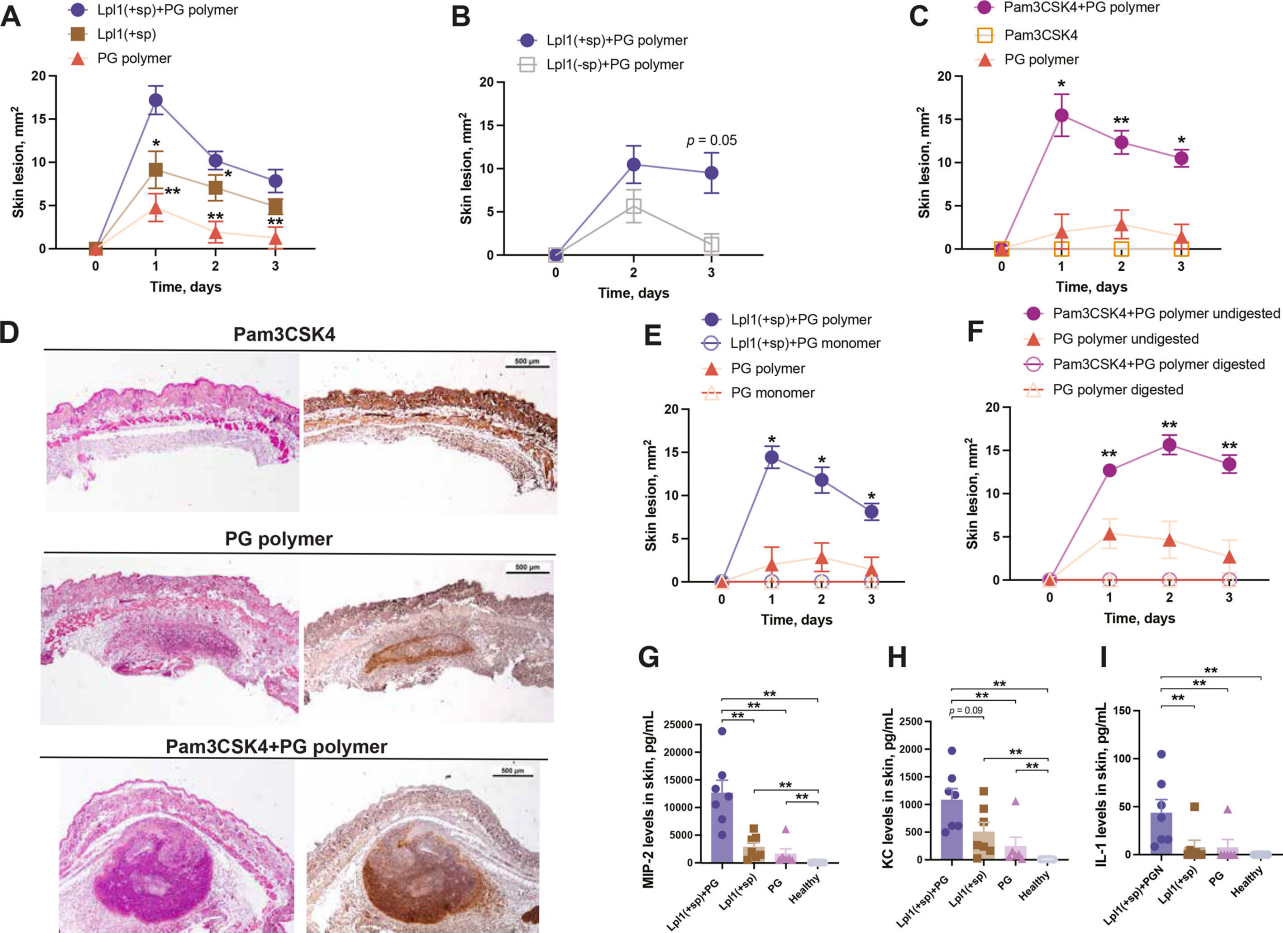
Co-injection of *S. aureus* Lpp and PG exerts a synergistic inflammatory effect in mouse skin. The skin lesion sizes (mm^2^) in NMRI mice (*n* = 6–7/group) up to 3 days after subcutaneous (s.c.) injection with 20 μL of (**A**) purified *S. aureus* lipoprotein, denoted as Lpl1(+sp) (2.5 μg/site, *n* = 7), purified *S. aureus* macromolecular peptidoglycan (PG polymer) (10 μg/site, *n* = 6) or co-injection of Lpl1(+sp) (2.5 μg/site) and PG polymer (10 μg/site, *n* = 7); (**B**) co-injection of PG polymer (10 μg/site) with either Lpl1(+sp) (2.5 μg/site, *n* = 4) or unlipidated Lpl1 protein, denoted as Lpl1(−sp) (2.5 μg/site, *n* = 4); and (**C**) Pam3CSK4 lipopeptide (1 μg/site, *n* = 4), PG polymer (10 μg/site, *n* = 4), or co-injection of Pam3CSK4 (1 μg/site) and PG polymer (10 μg/site, *n* = 5). (**D**) Corresponding representative photomicrographs of the mouse skin lesions of injected Pam3CSK4 (upper panel), PG polymer (middle panel), or combined Pam3CSK4 and PG polymer (lower panel) on day 3, with hematoxylin and eosin staining (left panels) or immunohistochemical fibrin staining (right panels). The skin lesion size (mm^2^) in NMRI mice up to 3 days after s.c. skin injection with 20 μL of (**E**) PG polymer (10 μg/site, *n* = 4) or purified monomeric fraction of PG (PG monomer) (10 μg/site, *n* = 4), or co-injections of Lpl1(+sp) (1 μg/site) with either PG polymer (10 μg/site, *n* = 4) or PG monomer (10 μg/site, *n* = 4); and (**F**) purified digested (PG polymer digested) (5 μg/site, *n* = 6) or undigested (PG polymer undigested) (5 μg/site, *n* = 6) forms or co-injection of Pam3CSK4 (1 μg/site) with either PG polymer digested (5 μg/site, *n* = 6) or undigested PG polymer (5 μg/site, *n* = 6). The levels of (**G**) macrophage inflammatory protein-2 (MIP-2), (**H**) keratinocyte chemoattractant (KC), and (**I**) IL-1β in the supernatants of skin biopsy homogenates from healthy (*n* = 5) NMRI mice or on day 3 after s.c. skin injection with 20 μL of Lpl1(+sp) (2.5 μg/site, *n* = 7), PG polymer undigested (PG) (10 μg/site, *n* = 6), or a combination of Lpl1(+sp) (2.5 μg/site) and PG polymer undigested (10 μg/site, *n* = 7). The data were pooled from two independent experiments. Statistical evaluations were performed using the Mann-Whitney *U*-test, with the data expressed as the mean ± standard error of the mean (**A–C, E, and F**), or with the data presented in a scatterplot with the mean ± standard error of the mean (**G–I**). *P < 0.05; **P < 0.01.

Increasing evidence suggests that *S. aureus* Lpp play an important pathogenic role in *S. aureus*-induced skin infection (13). Given that multiple *S. aureus*-associated molecular patterns may interact synergistically to enhance immune activation (9), we investigated whether purified *S. aureus* Lpp (specifically, Lpl1) would amplify skin inflammation when combined with *S. aureus* PG. To test this, mice were injected s.c. with Lpl1 (2.5 μg/site), polymeric peptidoglycan (PG polymer, 10 μg/site) purified from the *S. aureus* SA113Δ*lgt* mutant that lacks lipidated lipoproteins (14), or a combination of Lpl1 and PG polymer at the same doses. Notably, co-injection of Lpl1 and PG polymer resulted in significantly larger skin lesions than the injection of either component alone. This synergistic effect was evident from days 1 and 2 post-injection, compared with Lpl1 injection alone, and the lesion sizes remained significantly larger than those seen in the PG polymer control group throughout the study (Fig. 1A). No skin lesion was observed when the same volume of phosphate-buffered saline (PBS) was injected subcutaneously. Lpl1 induced larger skin lesions with scab formation, whereas PG polymer induced small, raised abscesses (Fig. S1).

To determine whether the lipid moiety of Lpp is critical for this synergy, mice were challenged with PG polymer (10 μg) combined with either intact Lpl1(+sp), containing the lipid moiety, or delipidated Lpl1(−sp). Mice that received the Lpl1(+sp)/PG mixture developed markedly larger lesions by day 3 post-injection compared with those that received the Lpl1(−sp)/PG combination, indicating that the lipid portion contributes to the synergistic inflammatory response (Fig. 1B).

To confirm this finding, a synthetic triacylated lipopeptide that mimics the lipid moiety of bacterial Lpp (Pam3CSK4; EMC, Tübingen, Germany) was co-injected with PG polymer. Mice that were injected with 1 μg of Pam3CSK4 plus 10 μg of PG polymer developed significantly larger skin lesions throughout the experimental period, compared with mice treated with either compound alone (Fig. 1C). Histological analysis on day 3 post-injection revealed that the skin tissues from the Pam3CSK4/PG polymer-injected group had extensive inflammation and fibrin deposition, whereas tissues from the control groups showed minimal or no inflammation (Fig. 1D). Our data clearly demonstrated *S. aureus* lipoproteins and PG synergistically induce skin lesions.

### Polymeric peptidoglycan mediates synergistic inflammation in the skin

To determine whether structural variations in *S. aureus* PG influence the inflammatory response in the skin, we compared the monomeric and polymeric PG fractions. PG monomer, an HPLC-purified monomeric fraction from the SA113Δ*lgt* mutant, failed to induce any visible skin inflammation when injected alone or in combination with Lpl1 (Fig. 1E). In contrast, PG polymer at the same concentration (10 μg/site) induced significantly larger skin lesions at all examined time points when co-administered with Lpl1 (1 μg/site), demonstrating that only the polymeric form exerts a synergistic inflammatory effect with Lpp (Fig. 1E).

To dissect further the inflammatory component of PG polymer, we compared its undigested and enzymatically digested forms. Mice were injected with either the fully digested PG polymer or the intact PG polymer, both of which were purified from the SA113Δ*lgt* mutant. Notably, the digested PG polymer, regardless of whether it was administered alone or with Pam3CSK4, failed to induce skin lesions. In contrast, undigested PG polymer (5 μg) combined with Pam3CSK4 (1 μg) retained its full inflammatory activity (Fig. 1F). These findings indicate that the intact polymeric structure is required for synergy with the lipid moieties of Lpp or synthetic lipopeptides. Consequently, the undigested PG polymer was used in all subsequent experiments.

To assess the local inflammatory response, the levels of neutrophil-attracting chemokines (macrophage inflammatory protein-2 [MIP-2] and KC), monocyte-attracting chemokines (MCP-1), and key cytokines involved in *S. aureus* skin infections (IL-17, IL-1β, and TNF-α) were measured in the skin homogenates. Mice that were injected with a mixture of Lpl1 (2.5 μg) and PG polymer (10 μg) displayed significantly higher MIP-2 levels than those receiving either compound alone (Fig. 1G). Furthermore, the levels of KC and MCP-1 were significantly higher in the mixture group compared to the PG polymer group, although not compared to the Lpl1 alone group (Fig. 1H; Fig. S2A). IL-1β levels were not significantly elevated in skin injected with either compound alone compared to PBS-injected healthy controls; however, they were significantly higher in the combination group than in both single-compound groups and healthy skin (Fig. 1I). No significant differences in TNF-α levels were detected between the groups (Fig. S2B), and IL-17 was not detectable in the skin homogenates. These results support the notion of a synergistic inflammatory mechanism for *S. aureus* skin infection, characterized by enhanced local production of neutrophil-attracting chemokines and IL-1β.

### *S. aureus* Lpp/PG mixture-induced skin inflammation is mediated by neutrophils and monocytes via TLR2 and NOD2

**Fig 2:**
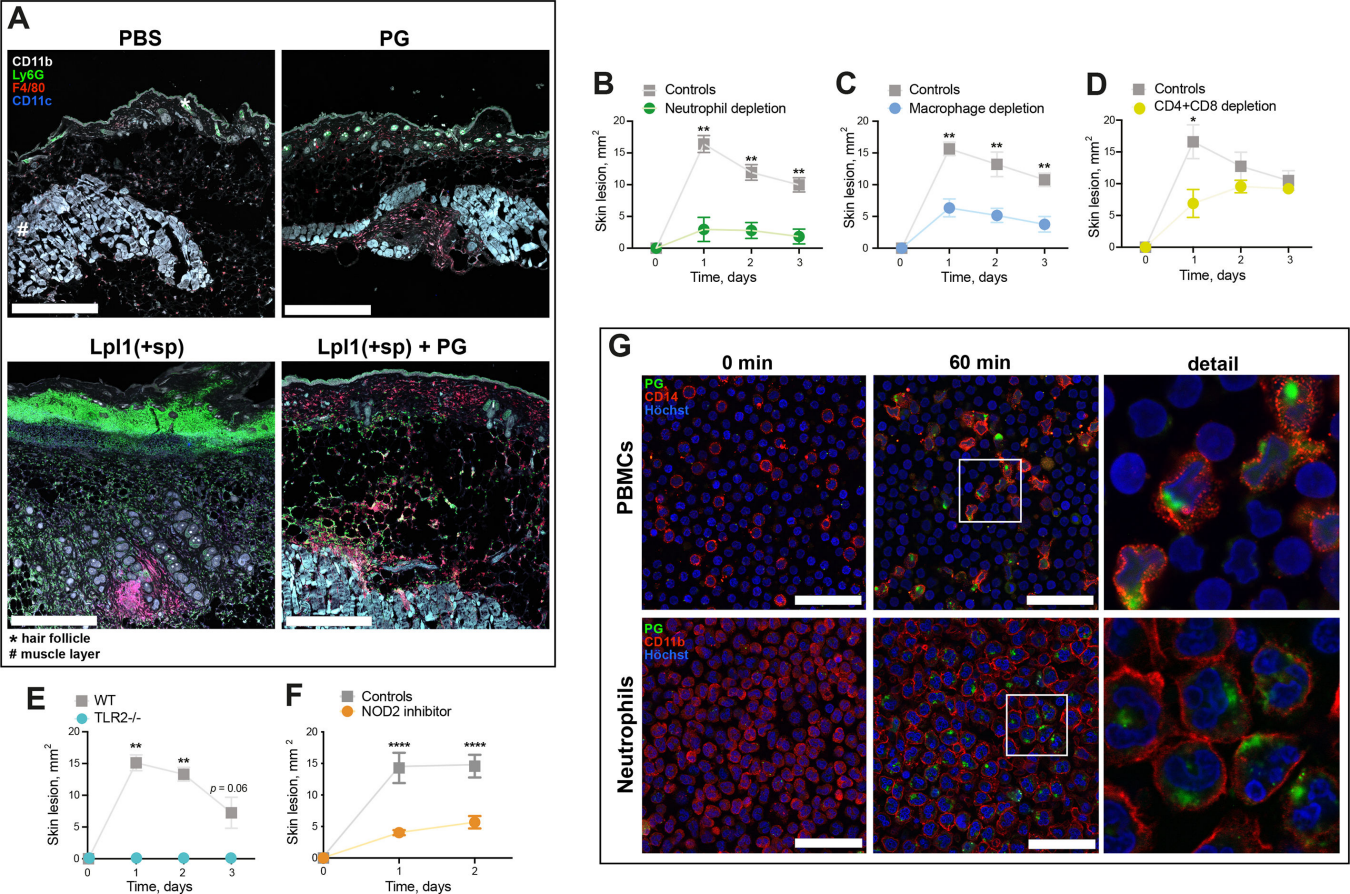
*S. aureus* Lpp/PG-induced skin inflammation is mediated by neutrophils and monocytes via TLR2 and NOD2. (**A**) Histological analysis of skin lesions by confocal imaging. Sections were stained for the myeloid marker CD11b, the neutrophil marker Ly6G, the macrophage marker F4/80, and the dendritic cell marker CD11c. Scale bar = 500 µm; * indicates autofluorescence of hair follicles; # indicates autofluorescence of muscle layer. (**B–D**) The skin lesion sizes (mm^2^) up to 3 days after s.c. co-injection with 20 μL of purified *S. aureus* lipoprotein, denoted as Lpl1(+sp) (1 μg/site), and purified *S. aureus* peptidoglycan polymer (PG) (10 μg/site) in NMRI mice that were (**B**) depleted of neutrophils using the anti-Ly6G antibody (*n* = 6/group); (**C**) depleted of monocytes using clodronate liposomes (*n* = 6/group); and (**D**) depleted of T cells using anti-CD4 and anti-CD8 antibodies simultaneously (*n* = 6/group). (**E**) C57BL/6 wild-type (WT) and TLR2-deficient (TLR2-/-) mice (*n* = 6/group). (**F**) NMRI mice treated with NOD2 inhibitor or vehicle (*n* = 6/group). The data were pooled from two independent experiments. Statistical evaluations were performed using the Mann-Whitney *U*-test, with data expressed as the mean ± standard error of the mean (**B–F**). *P < 0.05; **P < 0.01, ****P < 0.0001. (**G**) PG uptake by monocytes and neutrophils was validated with confocal microscopy. To identify monocytes and confirm intracellular uptake, PBMCs were counterstained with CD14. Purified peripheral neutrophils were counterstained with CD11b to define the intracellular space. PBMCs: peripheral blood mononuclear cells, Scale bar = 50 µm

To identify the immune cells that contribute to *S. aureus* Lpp/PG-induced skin abscess formation, skin biopsies of the lesion were sectioned and stained for neutrophils (Ly6G: green), macrophages (F4/80: red), dendritic cells (CD11c: blue), and the common myeloid marker CD11b (white). Compared with the PBS-treated biopsy (Fig. 2A, upper left panel), PG polymer (10 μg) alone induced mild macrophage infiltration into the dermis with an accumulation of macrophages observed in the subcutis (Fig. 2A, upper right panel). In the Lpl1(+sp) (1 μg)-treated skin, the predominant infiltrating immune cells were neutrophils. In contrast to PG polymer-only treatment, Lpl1(+sp) treatment often resulted in scab formation as seen in Fig. S1, which was strongly positive for neutrophil marker Ly6G (Fig. 2A, lower left panel). Treatment with a mixture of both PG polymer and Lpl1(+sp) showed a mixed phenotype of infiltrating neutrophils and macrophages (Fig. 2A, lower right panel).

To verify the importance of infiltrating neutrophils and monocytes for the abscess formation, NMRI mice were depleted of specific cell populations and then co-injected s.c. with Lpl1(+sp) (1 μg) and PG polymer (10 μg). Neutrophil depletion using the anti-Ly6G antibodies significantly reduced lesion size throughout the course of the experiment, compared to the isotype control-treated mice (Fig. 2B). Similarly, depletion of infiltrating monocytes using clodronate liposomes resulted in a marked reduction in lesion severity (Fig. 2C). These findings suggest that both of these cell types are active in mediating the inflammatory response.

To assess the role of T cells, mice were injected intraperitoneally (i.p.) with anti-CD4 and anti-CD8 antibodies to deplete CD4^+^ and CD8^+^ T cells, respectively. A significant reduction in lesion size was observed only on day 1 post-injection, and no differences were noted at later time points compared to the isotype controls (Fig. 2D), suggesting a limited role for T cells in sustained lesion development.

To determine whether the inflammatory response is mediated by TLR2, TLR2-knockout (TLR2^−/−^) and wild-type (WT) mice were co-injected with the Lpl1(+sp)/PG polymer mixture. Notably, the TLR2^−/−^ mice failed to develop skin abscesses, while the WT controls exhibited significantly larger lesions, especially during the first 2 days post-injection (Fig. 2E), indicating that the response is TLR2-dependent.

To understand if the abscess formation is also based on PG polymer-induced NOD2 signaling, mice were treated systemically with a specific NOD2 inhibitor during Lpl1(+sp)/PG polymer mixture-induced abscess formation. Indeed, NOD2 inhibition significantly reduced skin lesion size on days 1 and 2 after challenge (Fig. 2F).

To better understand the impact of PG polymer on immune activation, isolated peripheral blood monocytic cells (PBMCs) and neutrophils were challenged to phagocytose fluorescently labeled PG polymer (PG: green). To visualize the intracellular space and identify monocytes, PBMCs were counterstained with CD14 (red). In contrast to 0 min of incubation, at 60 min, monocytes had engulfed PG (Fig. 2G, upper panel). The same was observed for isolated peripheral neutrophils counterstained with CD11b (red) to visualize the intracellular space (Fig. 2G, lower panel).

### Fibrinogen depletion abrogates Lpp/PG mixture-induced skin inflammation

**Fig 3:**
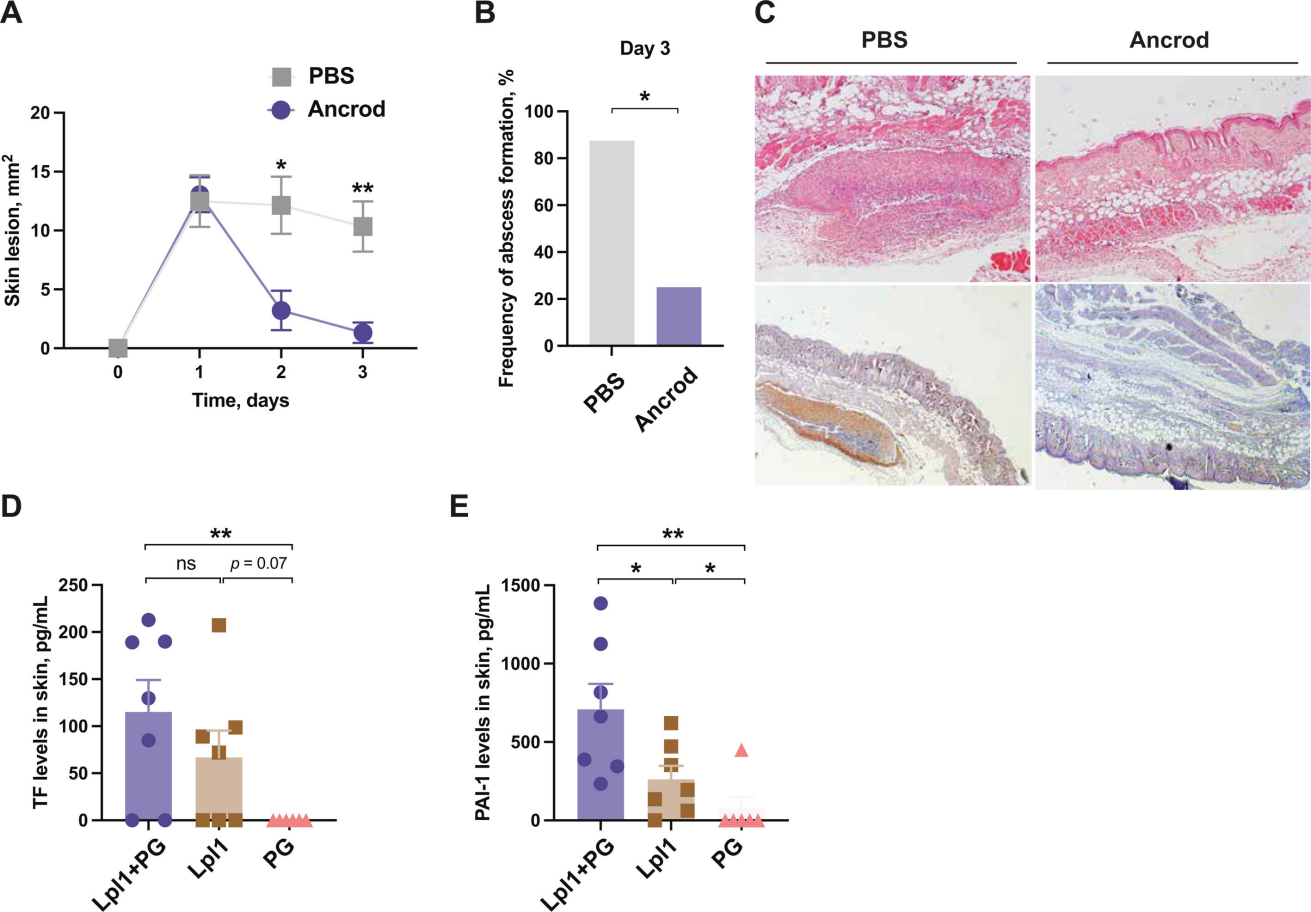
Fibrinogen depletion abrogates the skin inflammation induced by *S. aureus* Lpp and PG. NMRI mice depleted of fibrinogen using Ancrod or treated with PBS as a control (*n* = 8/group) up to 3 days after s.c. co-injection with 20 μL of purified *S. aureus* lipoprotein, denoted as Lpl1 (1 μg/site) and purified *S. aureus* macromolecular peptidoglycan undigested (PG) (10 μg/site) to assess (**A**) the skin lesion size (mm^2^), and (**B**) the frequency of skin abscess formation on day 3, with (**C**) the corresponding representative photomicrographs of the skin lesions in mice co-injected with Lpl1 and PG and treated with PBS (left panels) or Ancrod (right panels) on day 3, stained with hematoxylin and eosin (upper panels), or immunohistochemical staining for fibrin (lower panels). The levels of (**D**) tissue factor (TF) and (**E**) plasminogen activator inhibitor-1 (PAI-1) in the supernatants of NMRI skin biopsy homogenates on day 3 after s.c. injection with 20 μL of Lpl1 (2.5 μg/site, *n* = 7), PG (10 μg/site, *n* = 6), or co-injection of Lpl1 (2.5 μg/site) and PG (10 μg/site, *n* = 7). The data were pooled from two independent experiments. Statistical evaluations were performed using the Mann-Whitney *U*-test, with the data expressed as the mean ± standard error of the mean (**A**), or Fisher’s exact test (**B**), or the Mann-Whitney *U*-test, with the data presented as a scatterplot with the mean ± standard error of the mean (**D and E**). *P < 0.05; **P < 0.01; ns = not significant.

To study the role of coagulation in the synergistic inflammatory effect induced by the combination of *S. aureus* Lpp and PG, fibrinogen depletion was performed through Ancrod treatment in the murine skin inflammation model. On day 1 post-injection, skin lesion severity was similar in the fibrinogen-depleted and control mice. However, lesion resolution was significantly accelerated in fibrinogen-depleted mice, with nearly complete disappearance of lesions by day 2 and significant differences being observed on both days 2 and 3 (Fig. 3A). Histopathological analysis revealed that >80% of the injection sites in the control mice developed abscesses, whereas only 20% of the injection sites in the fibrinogen-depleted mice did so (Fig. 3B). Representative histological images demonstrated well-formed abscess-like structures with fibrin(ogen)-positive capsules in the control mice, which were absent in the Ancrod-treated mice on day 3 post-injection (Fig. 3C).

To explore whether Lpp/PG co-injection enhances coagulation and inhibits fibrinolysis, the tissue levels of tissue factor (TF) and plasminogen activator inhibitor-1 (PAI-1) were measured. Lpp/PG co-injection induced higher TF levels compared to PG alone (Fig. 3D) and significantly increased the PAI-1 levels compared to injection of either Lpp or PG alone (Fig. 3E).

### Combined disruption of lipidation and peptidoglycan O-acetylation attenuates *S. aureus*-induced skin infection

**Fig 4:**
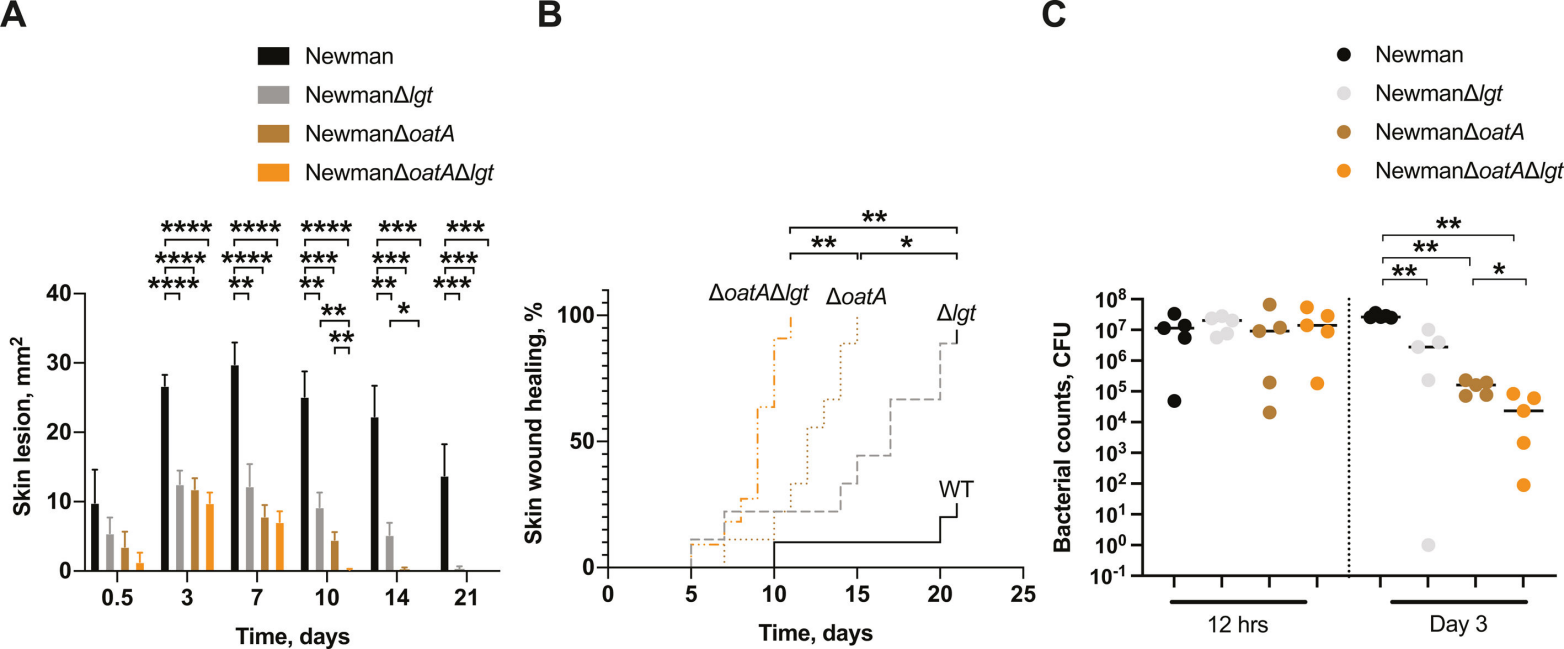
Combined abrogation of lipidation and peptidoglycan O-acetylation attenuates *S. aureus*-induced skin infection. The skin lesion sizes (mm^2^) (**A**), and frequencies of skin wound healing (**B**) in NMRI mice (*n* = 9–11/group) up to 21 days after s.c. injection with 50 μL of *S. aureus* Newman parental strain, NewmanΔ*lgt* mutant strain, NewmanΔ*oatA* mutant strain, or NewmanΔ*oatA*Δ*lgt* double-mutant strain (4 × 10^6^ colony-forming units [CFU]/site). Bacterial counts in the supernatants of skin biopsy homogenates at (**C**) 12 h and 3 days after s.c. infection with the Newman parental strain, NewmanΔ*lgt* mutant strain, NewmanΔ*oatA* mutant strain, or NewmanΔ*oatA*Δ*lgt* double-mutant strain (4 × 10^6^ CFU/site) in NMRI mice (*n* = 5/group). The data were pooled from two independent experiments. Statistical evaluations were performed using the Mann-Whitney *U*-test, with the data expressed as the mean ± standard error of the mean (**A**), or presented as a scatterplot with a line indicating the median value (**C**). Statistical evaluations were performed using the Mantel-Cox log-rank test (**B**). *P < 0.05; **P < 0.01; ***P < 0.001; ****P < 0.0001.

A *S. aureus* strain lacking lipoprotein lipidation exhibits diminished lipoprotein-induced inflammatory responses (8, 9). In addition, loss of peptidoglycan O-acetylation affects its structural properties and susceptibility to host recognition and degradation (15). To validate the findings in a clinically relevant context, we used an *S*. *aureus* strain lacking both lipidation (Δ*lgt*) and peptidoglycan O-acetyltransferase (Δ*oatA*) to induce skin infection in mice. This double-mutant was compared with the WT parental strain and the respective single mutants in the murine model. Mice that were infected with the double-mutant exhibited the smallest skin lesions throughout the infection course (Fig. 4A), and these mice had the shortest resolution time of 10 days, compared to 15 days for the mice with the Δ*oatA* mutant and 20 days for the mice with the Δ*lgt* mutant. In contrast, the lesions caused by the WT Newman strain showed only 20% resolution by day 21, with a median lesion size of 15 mm² at the experiment’s end (Fig. 4A and B). Importantly, the bacterial loads in skin biopsies taken on day 1 post-infection were comparable across all the groups. However, by day 3, the mice infected with the double-mutant exhibited the lowest bacterial burden, whereas the WT had the highest burden, and the single-mutants showed intermediate levels (Fig. 4C).

### Neutrophils, but not monocytes or coagulation, mediate the attenuated virulence of the *S. aureus* Δ*lgt*Δ*oatA* mutant

**Fig 5:**
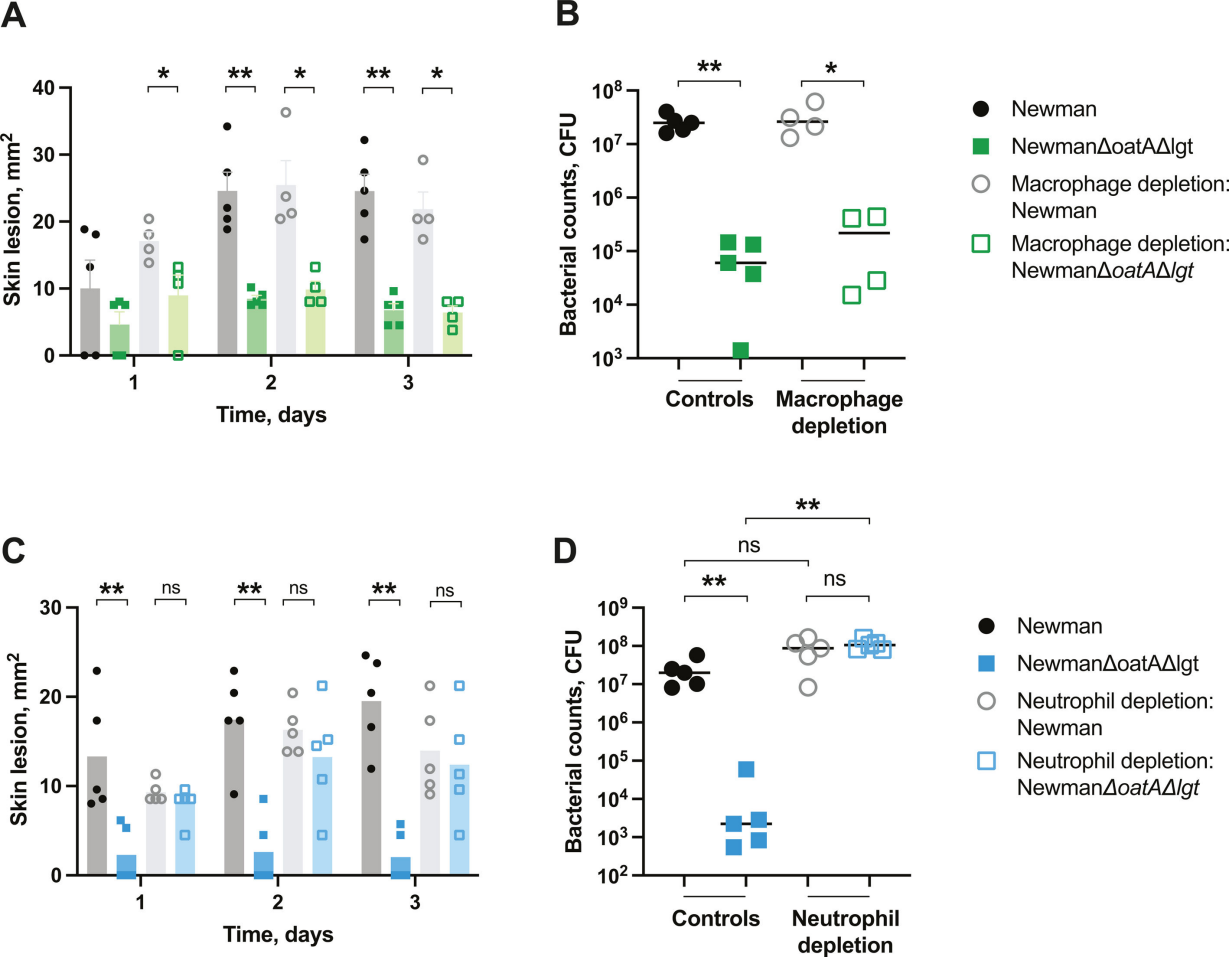
Neutrophils mediate the attenuated virulence of the *S. aureus* Δ*lgt*Δ*oatA* mutant in the *S. aureus* skin infection model. The skin lesion sizes (mm^2^) up to 3 days (**A**) and bacterial counts in the supernatants of skin biopsy homogenates on day 3 (**B**) after s.c. injection with 50 μL of the *S. aureus* Newman parental strain or the NewmanΔ*oatA*Δ*lgt* double-mutant strain (4 × 10^6^ CFU/site) in NMRI mice depleted of monocytes using clodronate liposomes or treated with PBS control liposomes as a control (*n* = 4 or 5/group). The skin lesion sizes (mm^2^) up to 3 days (**C**) and bacterial counts in the supernatant of skin biopsy homogenates on day 3 (**D**) after s.c. infection with the Newman parental strain or the NewmanΔ*oatA*Δ*lgt* double-mutant strain (4 × 10^6^ CFU/site) in NMRI mice depleted of neutrophils using the anti-Ly6G antibody or treated with the isotype control (*n* = 5/group). Statistical evaluations were performed using the Mann-Whitney *U*-test, with the data presented as a scatterplot with the mean ± standard error of the mean (**A and C**) or presented as a scatterplot with a line indicating the median value (**B and D**). *P < 0.05; **P < 0.01; ns = not significant.

Having established the critical roles of neutrophils, macrophages, and coagulation in Lpp/PG-induced skin inflammation, we next evaluated their contributions in the skin infection model using an *S. aureus* strain that lacks both lipidation (Δ*lgt*) and peptidoglycan O-acetyltransferase (Δ*oatA*). Monocyte depletion had no discernible effect on either skin lesion size or bacterial load in the skin biopsies (Fig. 5A and B). Similarly, fibrinogen depletion did not affect either lesion size or bacterial burden (Fig. S4A and B). In contrast, neutrophil depletion completely abolished the differences in lesion size and bacterial load between the mice infected with the WT strain and those infected with the double-mutant strain (Fig. 5C and D). These findings indicate that neutrophils are the key mediators of the attenuated skin infection phenotype observed for the Δ*lgt*Δ*oatA* mutant.

## DISCUSSION

In this study, we demonstrate that subcutaneous injection of *S. aureus* Lpp and PG synergistically induce skin abscesses in mice. Both the lipid moieties of Lpp and the polymeric structure of PG are essential for this effect. Notably, neutrophils and monocytes are key contributors to abscess formation, with TLR2 and NOD2 playing a crucial role in mediating this response. Importantly, fibrinogen depletion effectively prevents the abscess formation caused by the Lpp/PG mixture, highlighting the potential involvement of coagulation and fibrinolysis in these skin lesions. The *S. aureus* strain that lacks both lipidation and peptidoglycan *O*-acetyltransferase exhibited significantly reduced virulence, leading to quicker wound healing compared to the corresponding single-mutant strains, supporting the findings we obtained with purified molecules. The attenuation of virulence in the double-mutant was completely reversed by neutrophil depletion, suggesting the essential role of neutrophils in the synergistic effect of Lpp and PG in *S. aureus*-induced skin infections.

We demonstrate that the combined application of Lpl1(+sp) and PG induces a 5-fold to 10-fold increase in MIP-2 levels in the skin, compared to each ligand alone targeting the TLR2 or NOD2 immune receptors. This pronounced response reflects a clear synergistic effect, approaching the magnitude of the cytokine storm typically associated with staphylococcal superantigens (SAgs). For example, the SAg enterotoxin B (SEB) significantly upregulates MIP-2 expression in mice, thereby amplifying the inflammatory response. When SEB is injected into murine air pouches, it triggers a time-dependent secretion of MIP-2, with levels peaking 1 h after the administration of 10 μg SEB. This rapid chemokine surge plays a critical role in neutrophil recruitment (16). Moreover, the expression of MIP-2 following *S. aureus* infection is differentially regulated across mouse strains. C57BL/6 mice exhibit a marked increase in MIP-2 expression at 4 h post-infection, whereas A/J mice showed no such response (17). Interestingly, in our experiments, we did not observe any major qualitative differences in lesion development between NMRI and C57BL/6 mice under the tested conditions. The overall patterns of inflammation and lesion formation were comparable.

The potent induction of MIP-2 by the combined action of lipidated lipoproteins and PG is probably attributable to their simultaneous stimulation of the immune receptors TLR2 and NOD2. This can trigger a similar severe cytokine storm as that observed with SAgs that activate a large fraction of T cells through cross-linking of the major histocompatibility complex (MHC) class II molecules with T-cell receptors (18). High levels of MIP-2 contribute to disease pathogenesis by promoting excessive neutrophil infiltration and sustained inflammation. Consequently, therapeutic strategies that target MIP-2 or its associated signaling pathways may have the potential to mitigate inflammatory damage. The specific conditions under which *S. aureus* simultaneously releases Lpp and PG to trigger a cytokine storm remain unclear. However, β-lactam antibiotics, which inhibit cell wall biosynthesis, may facilitate the concurrent release of both components. This hypothesis warrants further investigation in future studies.

It appears that the synergistic interaction of bacterial PG with other bacterial components in disease induction during infections is a common phenomenon. Lipopolysaccharide (LPS), a gram-negative bacterial cell wall component, synergizes with PG to induce TNF-α synthesis in the human monocytic cell line Mono Mac 6 (19). Similar synergistic effects of LPS and PG have been observed in osteoclast formation *in vitro* (20). *In vivo*, PG has been shown to enhance bone resorption in mouse gingiva when co-injected with LPS (21), indicating that gram-positive PG and gram-negative LPS work together to induce osteoclastogenesis in periodontitis, and these two components might well be present during a mixed infection. Furthermore, it has been shown that PG and bacterial DNA synergistically induce the production of coagulation factors, such as TF, in human PBMCs (22). In addition, PG and lipoteichoic acid (LTA) have been demonstrated to act synergistically, leading to respiratory failure and septic shock in pigs (23). Interestingly, Lpp can also exert synergistic effects with other bacterial components. For example, PG-associated Lpp from gram-negative bacteria is shed into the serum and, in combination with LPS, synergistically activates macrophages to release IL-6 and produce nitrite (20).

Polymers, but not monomers, of PG synergistically induce skin lesions in combination with Lpp. Our data show that subcutaneous injection of PG polymers results in small skin lesions with microscopic abscess formation, whereas the injection of PG monomers fails to elicit any skin inflammation. This is in line with previous studies demonstrating that PG polymers are significantly more potent than monomeric PG or muramyl dipeptide (MDP) with respect to triggering proinflammatory cytokines in human monocytes and neutrophils (24). PG is sensed by the intracellular pattern recognition receptors NOD1 and NOD2, which recognize gram-negative and gram-positive PG, respectively. These NOD receptors are primarily localized in the cytoplasm, where they diffuse freely and monitor the intracellular environment for signs of bacterial infection (25). A minor fraction of NOD receptors is associated with the plasma membrane or recruited to endosomal or phagosomal membranes, particularly following pathogen uptake. This membrane association is facilitated by lipid modifications such as S-palmitoylation (26). Since NOD receptors are mainly intracellular, PG must be internalized into host cells, predominantly through endocytosis. We propose that larger PG fragments are more efficiently taken up than monomeric PG, which may explain the stronger immune stimulation by PG polymers compared to monomers (9).

In *S. aureus*, PG is O-acetylated by the enzyme PG O-acetyltransferase A (OatA). This modification renders the bacterium resistant to lysozyme, which is produced by immune cells (e.g., neutrophils and macrophages), as well as epithelial cells (15). Lysozyme is a key component of the innate immune system and provides nonspecific defense against bacterial pathogens. oatA mutants are not only lysozyme-sensitive and less virulent, but they also induce a strong activation of the NLRP3 inflammasome and IL-1β secretion (27). This inflammasome activation is likely driven by PG fragments generated through lysozyme-mediated degradation.

As previously discussed (9), although both Lpp and PG ultimately activate NF-κB, they do so via distinct signaling pathways: Lpp signals through the TLR2–MyD88 axis, while PG signals via the NOD2–RIP2 pathway. Because these pathways use different adaptor molecules, there is no expected competition. This would typically result in an additive effect; however, a synergistic response suggests additional cross-talk. We hypothesize that initial activation of the TLR2–MyD88 pathway by Lpp, whose ligand-binding domain is immediately accessible, induces TNF-α production, which, in turn, upregulates NOD2 expression. This increased NOD2 abundance enhances cellular responsiveness to PG (28, 29), thus explaining the observed synergy between Lpp and PG polymers. A relevant question is whether the levels of Lpp and PG used in our studies reflect the physiological concentrations present during *S. aureus* skin infection. Although it is impossible to precisely determine the concentrations of these components in local skin tissue during infection, PG is the most abundant structural component of the bacterial cell wall, and bacterial loads in localized abscesses can be very high. Therefore, it is highly possible that the amounts of Lpp and PG administered in our experiments are within the physiological range encountered during infection.

Our data suggest that both monocytes and neutrophils play significant roles in the synergistic effects of PG and Lpp. It is well established that *S. aureus* lipoproteins and lipopeptides can rapidly induce the release of neutrophil chemokines, such as MIP-2 and KC, from tissue-resident macrophages via TLR2 signaling (7). However, despite this release of chemokines, a single injection of lipopeptides (Pam3) did not result in any visible skin lesions, indicating that neutrophil infiltration alone is insufficient to trigger local skin inflammation. Pure PG preparations have been shown to induce proinflammatory cytokine release from innate immune cells (30), in a process that requires PG internalization and lysosomal trafficking (31). Importantly, nucleotide oligomerization domain (NOD)-like receptors, which are intracellular receptors, recognize PG monomers, but not polymers (32, 33). NOD2 deficiency led to delayed, albeit exacerbated, skin lesions in the *S. aureus* skin infection model, and the action of NOD2 is dependent upon IL-1β-amplified production of IL-6 and consequent neutrophil activation (34). Our findings support a model that explains the synergy between PG and Lpp in skin infections: Lpp trigger chemokine release from the resident macrophages in the skin tissue, leading to neutrophil infiltration. Polymeric PG is then internalized by these neutrophils and infiltrating monocytes, transported to the lysosome, and degraded into monomeric PG, which is recognized by NOD receptors. This recognition enhances the inflammatory process, leading to further chemokine release and creating a positive feedback loop that accelerates the inflammation.

Fibrin is the major component of abscess capsules and pseudocapsules (35). Local fibrinolytic activation by the staphylokinase produced by *S. aureus* is known to reduce the disease severity of *S. aureus* skin infection (36). Previous studies have shown that Lpp expression in *S. aureus* disrupts the balance between coagulation and fibrinolysis in local skin, promoting abscess formation in a skin infection model (13). Moreover, *S. aureus* PG is able to induce TF expression and procoagulant activity in human monocytes and vascular endothelial cells (37, 38). Here, we show that Lpp and PG work synergistically to (i) enhance coagulation by increasing TF expression and (ii) suppress fibrinolysis by increasing the PAI-1 levels in the local skin environment. This imbalance contributes significantly to abscess formation. Notably, fibrinogen depletion mediated by Ancrod significantly reduced the sizes of the skin lesions induced by Lpp/PG injection, further supporting the idea that the local coagulation/fibrinolysis imbalance is crucial for the development of Lpp/PG-induced skin lesions.

*S. aureus* resists lysozyme, which is a key antimicrobial enzyme secreted by neutrophils and macrophages, owing to O-acetylation of the N-acetylmuramic acid in its peptidoglycan (15). This modification, which is mediated by the O-acetyltransferase (OatA), makes the PG less-susceptible to lysozyme degradation. Mutants that lack OatA are sensitive to lysozyme and, consequently, exhibit reduced virulence (39). Although using a double mutant deficient in both OatA and Lgt is not ideal, it is the best available model, as *S. aureus* cannot survive without cell wall peptidoglycan. Nevertheless, our investigation of this double mutant in the skin infection model clearly demonstrates that both peptidoglycan and lipoproteins contribute significantly to the pathogenesis of *S. aureus* skin infections.

In summary, our findings demonstrate that PG and Lpp in *S. aureus* work synergistically to induce skin abscess formation by triggering the coagulation cascade and inhibiting fibrinolysis. Notably, *S. aureus* strains that are deficient in both lipidation and peptidoglycan O-acetyltransferase exhibit reduced virulence, resulting in smaller skin lesions and faster wound healing through a process that is entirely dependent upon neutrophils. This study offers critical new insights into how *S. aureus* components contribute to skin lesion formation in SSTIs. Understanding these mechanisms could lead to the development of novel therapeutic strategies that target this pathway.

## MATERIALS AND METHODS

### Mice

Female NMRI mice, aged 6–10 weeks, were purchased from Envigo (Venray, the Netherlands), while gender- and age-matched 6- to 10-week-old C57BL/6 wild-type (WT) mice and TLR2-deficient B6.129-Tlr2^tm1Kir^/J (TLR2^−/−^) mice were purchased from Charles River Laboratories (Sulzfeld, Germany) and The Jackson Laboratory (Bar Harbor, ME, USA), respectively. All mice were housed in the animal facility of the Department of Rheumatology and Inflammation Research, University of Gothenburg. Mice were kept under standard temperature and light conditions and were fed laboratory chow and water *ad libitum*.

### Expression and purification of Lpl1(+sp) and Lpl1(–sp)

The preparation and purification of *S. aureus* lipoproteins Lpl1(+sp) and Lpl1(−sp) were performed by Dr. Nguyen (Microbial Genetics, University of Tübingen, Germany), as previously described (40). Lpl1(+sp) was isolated from the membrane fraction of *S. aureus* SA113 (pTX30::lpl1-his), and Lpl1(−sp) was derived from the cytoplasmic fraction of SA113Δlgt (pTX30::lpl1(-sp)-his). Both proteins were purified using Ni-NTA affinity chromatography. For protein expression, bacteria were cultured aerobically at 37°C in BO-medium without xylose until they reached an optical density of 0.5 at 578 nm (OD_578_), followed by 4 h of culturing in a medium with 0.5% xylose. The cells were harvested, washed with Tris buffer (20 mM Tris, 100 mM HCl, pH 8.0), and resuspended in Tris buffer with protease inhibitors and lysostaphin (30 µg/mL) and then incubated at 37°C for 2 h. After ultracentrifugation (235,000 × *g* for 45 min at 4°C), the cytoplasmic proteins were collected. For membrane proteins, the pellet was dissolved in Tris buffer that contained 2% Triton X-100 overnight at 6°C, followed by centrifugation. Ni-NTA Superflow beads (Qiagen) were used for purification, with sequential washes in Tris buffer containing 0.25% Triton X-100 and 20 mM (and then 40 mM) imidazole. Elution was performed with 500 mM imidazole in Tris buffer. Proteins were concentrated by centrifugal ultrafiltration (10 kDa cutoff; Sartorius), dialyzed overnight at 6°C in DPBS (MWCO 6–8 kDa; Merck), and lyophilized. For each lyophilized sample, 2 µg was analyzed using SDS-PAGE. Purified Lpl1 was stored at −70°C and diluted in PBS before use. The purified compounds of Lpl1 were stored at −70°C until use and adjusted to the required concentration in PBS before each experiment.

### Purification of macromolecular PG (PG polymer)

The preparation and purification of the *S. aureus* macromolecular PG (PG polymer) were performed as previously described (41, 42) with some modifications. Polymeric PG was isolated from *S. aureus* SA113Δ*lgt*. Briefly, cells were grown until the cultures reached an OD_578_ of 0.6, after which the cells were harvested by centrifugation at 3,000 × *g* for 30 min, boiled in the presence of 5% SDS for 30 min, and disrupted with glass beads. Insoluble polymeric PG was harvested by centrifugation at 30,000 × *g* for 30 min, followed by washing several times with lukewarm water to remove the SDS. Broken cell walls were suspended in 100 mM Tris-HCl (pH 7.2), treated with 10 μg/mL DNase and 50 μg/mL RNase A for 2 h and, subsequently, with 100 μg/mL trypsin for 16 h at 37°C. To remove cell wall teichoic acid, the PG preparations were incubated with 48% hydrofluoric acid (HFA) for 48 h at 4°C. PG was harvested by centrifugation at 30,000 × *g* for 30 min and washed several times with water until the complete removal of HFA was achieved. Finally, the PG was resuspended in water and lyophilized.

### Preparation of PG monomer and polymer fractions

Purification of monomeric and polymeric PG fragments was performed as previously described (42). Isolated macromolecular PG was resuspended in 25 mM sodium phosphate buffer (pH 7.0) and digested with mutanolysin (Sigma-Aldrich) for 18 h at 37°C. The enzyme reaction was stopped by boiling the sample for 5 min at 90°C, and insoluble contaminants were removed by centrifugation. Separation of the PG monomer and polymer fractions was carried out by preparative HPLC on a reversed-phase column (ProntoSIL 120-3-C18 AQ 3 mm, NC 250 × 8 mm; Bischoff Analysentechnik und -geräte GmbH, Leonberg, Germany) using a linear gradient of 0.1% TFA in water and 0.1% TFA in 50% acetonitrile over 100 min at a flow rate of 2 mL/min. Peaks were detected in a spectrophotometer at 205 nm. Separation was carried out without reduction with borohydride to avoid structural modifications. The major monomer peak with retention time (RT) of 43 min was collected and desalted on the same column with a water-methanol gradient. In the same way, the polymeric fraction collected at RT 75-95 min was desalted on the same column with a water-methanol gradient. The purified PG monomer and PG polymer fractions were subsequently lyophilized.

One milligram of the monomer product was resuspended in phosphate buffer (pH 7.0), and 100 µL was then mixed with an equal volume of 0.5 M sodium borate buffer (pH 9.0) that contained freshly dissolved sodium borohydride (10 mg/mL) and reduced for 30 min at room temperature. Excess borohydride was deactivated by adding 20% phosphoric acid. To confirm appropriate digestion of the macromolecular PG, the reduced sample was analyzed by RP-HPLC using a Poroshell 120 EC-C18, 2.7-µm column (Agilent Technologies, Waldbronn, Germany) and a linear gradient of 5%–30% MeOH in 100 mM sodium phosphate buffer (pH 2.2) at a flow rate of 0.6 mL/min. Peaks were detected at 205 nm in a spectrophotometer (Fig. S5A). The purity of the PG monomer product was confirmed by HPLC analysis using the same column and buffer conditions, except that a steeper gradient was used to shorten the analysis time (Fig. S5B).

LPS contamination was routinely checked using the QCL 1000 chromogenic LAL endpoint assay (Lonza, Basel, Switzerland), and residual LPS was removed using the EndoTrap Blue Endotoxin Removal Kit (Profos AG, Regensburg, Germany). Thus, the LPS content of the final preparation was very low (0.005 EU/mg), and not sufficient to yield detectable activation of hTLR4-HEK293 cells transfected with an NF-κB-reporter plasmid (pNF-κB-TA-Luc), whereas stimulation of hTLR4-HEK293 cells with LPS routinely resulted in NF-κB activation. The final sample of the PGmonomer, which was of high purity (no LPS, Lpp, or LTA contamination) and with a mass of 1,238 [M + H]^+^, was used for the experiments.

### Preparation of bacterial gene deletions

Construction of single- and double-deletion mutants of *lgt* and *oatA* in *S. aureus* Newman was carried out using the pBASE6 plasmid (43). The 1 kb upstream and downstream regions of *lgt* and *oatA* were amplified using appropriate primers (Table S1), ligated with linearized pBASE6 (*Eco*RI restriction site) using the Hi-Fi DNA Assembly Master Mix (New England Biolabs, Ipswich, MA, USA), and transformed into chemically competent *E. coli* DC10B cells. The plasmid-containing colonies were picked, and the isolated plasmid was transformed into *S. aureus* Newman strains. Mutagenesis was conducted as previously reported (44). Mutants were confirmed using PCR for the detection of the gene deletion. All mutants exhibited growth rates comparable to the parent strain, indicating no growth defects (Fig. S3).

### Induction of skin inflammation with staphylococcal lipoproteins and peptidoglycan in a murine *S. aureus*-induced skin infection model

To study the roles of *S. aureus* Lpp and PG in murine skin inflammation and skin infection, six sets of experiments were performed using NMRI, C57BL/6 wild-type, and TLR2^−/−^ mice. The animals were anesthetized with ketamine hydrochloride (Pfizer AB, Stockholm, Sweden) and medetomidine (Orion Pharma, Espoo, Finland), their backs were shaved, and they were injected subcutaneously (s.c.) with one of the following compounds in 20 µL of PBS, as outlined below: (i) Lpl1(+sp), purified macromolecular PG (PG polymer), or mixtures of Lpl1(+sp) with PG polymer; (ii) purified Lpl1(+sp) or Lpl1(–sp) *S. aureus* Lpp together with PG polymer; (iii) synthetic lipopeptide Pam3CSK4, PG polymer, or mixtures of Pam3CSK4 and PG polymer; (iv) PG polymer, purified monomeric PG (PG monomer), or mixtures of Lpl1(+sp) with either PG polymer or PG monomer; (v) Pam3CSK4 mixed with either undigested or digested PG polymer, or digested/undigested PG polymer alone; and (vi) in 50 μL of PBS with live *S. aureus* Newman, NewmanΔ*lgt* mutant, NewmanΔ*oatA* mutant, or NewmanΔ*lgt*Δ*oatA* double mutant. The skin inflammation experiments lasted 3 days, whereas in the skin infection model, skin lesions and wound healing were monitored for up to 21 days. To assess potential synergistic effects, we used doses of Lpp and polymeric PG that individually induce only mild or subthreshold skin inflammation. This approach facilitates the detection of synergistic responses when the two stimuli are combined. Specifically, Lpl1 at 1–2.5 μg per site induced minimal skin lesions, whereas PG polymer at 10 μg per site elicited mild, but not fulminant, skin inflammation. The resulting skin lesions were measured with calipers until the mice were sacrificed. The sizes of the skin lesions were calculated using the mathematical formula for the area of an ellipse. Two observers (M.M. and T.J.) inspected the lesion sizes for each mouse in a blinded manner. A healed skin infection was defined as a skin wound that was fully covered by freshly healed epidermis and lacked clinical signs of tissue necrosis and granulation, inflammatory exudates, or skin abscess. At the end of the experiments, the mice were anesthetized with ketamine/medetomidine and sacrificed by cervical dislocation, and skin biopsies were collected post-mortem (45).

### *In vivo* cell depletion

Anti-Ly6G (clone 1A8; BioXCell), a specific monoclonal antibody (mAb), selectively depletes blood neutrophils in mice (46). NMRI mice were injected i.p. with one dose of 400 μg of anti-Ly6G or isotype control (clone 2A3; BioXCell) in 200 μL of PBS/mouse, 1 day prior to skin challenge with co-injections of Lpl1 and PG polymer, and 1 day after the challenge. The efficacy of *in vivo* neutrophil depletion with this protocol has been demonstrated in our previous studies (7, 47).

Clodronate liposomes (Liposoma BV, Amsterdam, the Netherlands) function as selective eliminators of monocytes/macrophages in mice (48). NMRI mice were intravenously (i.v.) injected with 200 μL of clodronate liposomes or PBS control liposomes (Liposoma) 1 day prior to skin challenge with Lpl1 together with PG, and 1 day after the challenge. This treatment efficiently depletes circulating monocytes, Kupffer cells in the liver, and macrophages in the spleen, while having no significant impact on neutrophils. This approach does not deplete residual macrophages or dendritic cells in local skin tissue. The efficacy of *in vivo* monocyte depletion with this protocol has been demonstrated in our previous studies (7, 47).

CD4 and CD8 T cells were depleted simultaneously using rat anti-mouse CD4 mAb (clone GK1.5; BioXCell) and rat anti-mouse CD8α mAb (clone 2.43; BioXCell) (7); the rat IgG2b isotype control mAb (clone LTF-2; BioXCell) served as the control. NMRI mice were i.p. injected with 1 dose of 400 μg of each antibody in 200 μL of PBS/mouse on the day before and the day after skin challenge with Lpl1/PG. The efficacy of *in vivo* T cell depletion with this protocol has been demonstrated in our previous studies (7).

### *In vivo* NOD2 inhibition

The NMRI mice were treated once a day with 10 µg/g body weight GSK717 in 200 µL of PBS (MedChemExpress, Monmouth, NJ, USA) or the same volume of PBS as controls (49). Treatment started 1 day prior to the induction of skin inflammation by mixtures of Lpl1(+sp) with PG polymer.

### *In vivo* fibrinogen depletion

Ancrod (Product No. 15/106; National Institute for Biological Standards and Control, South Mimms, UK), a thrombin-like enzyme derived from the snake venom of the Malayan pit viper Calloselasma rhodostoma, is known to deplete the fibrinogen levels *in vivo* (50, 51). NMRI mice were injected i.p. with either 200 μL of PBS as a control substance or with 2 units of Ancrod in 200 μL of PBS/mouse every 12 h. The treatment was initiated 12 h prior to the skin infection and continued until the experiment was terminated on day 3 in both the skin inflammation and skin infection models.

### Skin homogenate preparation and bacteriological examination of skin biopsies

At 12 h and on day 3 post-infection, the mice were euthanized, the skin was disinfected with 70% (vol/vol) ethanol, and skin biopsies encompassing the entire inflamed or infected area were acquired with a sterile 8-mm biopsy punch (Kai Medical, Seki, Japan), as previously described (13) and thereafter homogenized with TissueLyser II (Qiagen, Hilden, Germany) in 0.5 mL PBS. For the mice that received the purified compounds, the skin homogenates were centrifuged at 13,000 rpm for 10 min. The supernatants were collected and subsequently used for measurements of cytokines and chemokines. For the mice that were inoculated with live bacteria, the skin homogenate was diluted in PBS, spread on horse blood agar plates, and incubated for 24 h at 37°C. Viable counts of bacteria were performed and quantified as colony-forming units (CFUs). This method recovers approximately 85% of the bacteria present in a skin sample (45).

### Measurements of cytokine and chemokine levels in skin homogenates

The levels of macrophage inflammatory protein-2 (MIP-2), keratinocyte chemoattractant (KC), monocyte chemoattractant protein-1 (MCP-1), tumor necrosis factor alpha (TNF-α), IL-17, IL-1β, tissue factor (TF), and plasminogen activator inhibitor-1 (PAI-1) in the supernatant fluids from the skin homogenates were quantified using DuoSet ELISA kits (R&D Systems, Abingdon, UK), according to the manufacturer’s instructions.

### Immunofluorescence of skin biopsies

Skin biopsies were embedded in optimum cutting temperature compound (Thermo Fisher Scientific, Waltham, MA, USA) and flash-frozen. Frozen samples were cut into 10 μm sections using a Cryostar NX70 (Epredia, Portsmouth, NH, USA). Sections were stored at −80 degrees until further processing. Prior to staining, the sections were air-dried for 40 min, fixed in ice-cold acetone (Sigma Aldrich) for 8 min at –20 degrees, and air-dried for a further 15 min. Skin sections were circled with a barrier pen, rehydrated for 15 min in PBS, and blocked with 5% goat serum (Nordic Biolabs, Täby, Sweden) in PBS for 30 min. The unlabeled antibody against CD11c and the fluorescently conjugated antibodies CD11b-BV421, LY6G-FITC, and F4/80-APC (BioLegend, San Diego, CA, USA) were diluted in blocking buffer and added on the sections for overnight incubation at 4 degrees. The following day, sections were washed with PBS. The secondary antibodies, goat anti-hamster Alexa Fluor 568 (Invitrogen, Waltham, MA, USA), diluted in PBS, were added, and the sections were incubated in the dark for 40 min at room temperature. Sections were washed with PBS, counterstained with Hoechst 33342 (Thermo Fisher Scientific), and mounted with Prolong Diamond Antifade mount (Thermo Fisher Scientific). The sections were imaged with an SP8 confocal microscope (Leica, Wetzlar, Germany), and the images were stitched and smoothed with LAS X version 3.5.5 software (Leica). Brightness and contrast were adjusted using Adobe Photoshop version 24.6.0 (Adobe, San Jose, CA, USA).

### PG uptake experiment

Peripheral blood neutrophils and PBMCs were isolated from buffy coats obtained from healthy donors. In brief, leukocytes were separated by dextran sedimentation, followed by Ficoll-Paque density gradient centrifugation. PBMCs were collected from the Ficoll-Paque interface, while neutrophils were recovered from the pellet. Residual erythrocytes were removed by hypotonic lysis as described (52).

The cells were then resuspended in ice-cold Krebs–Ringer glucose phosphate buffer (KRG; 120 mM NaCl, 4.9 mM KCl, 1.7 mM KH_2_PO_4_, 8.3 mM Na_2_HPO_4_, 1.5 mM MgSO_4_, 10 mM glucose, and 1 mM CaCl_2_ in dH_2_O, pH 7.3). Cell purity and concentration were determined using a Sysmex KX-21N hematology analyzer (Sysmex Corporation, Kobe, Japan). Cells were kept on ice and used for experiments on the day of isolation.

The PG polymer was labeled with the Alexa Fluor 488 NHS ester dye (Invitrogen, Waltham, MA, USA) according to the manufacturer’s protocol. PBMCs and isolated neutrophils (89% purity) were pipetted on Super Frost Plus slides on ice and allowed to settle for 40 min. This was followed by a 30 min incubation with 20 µg/mL labeled PG on ice. For the 0 min time point, the cells were washed with ice-cold PBS and fixed in 4% phosphate-buffered formaldehyde for 10 min. For the PG uptake, the cells were transferred to 37°C for 60 min, followed by a washing step with PBS and fixation. For the staining, the cells were blocked with PBS with 5% goat serum (Nordic Biolabs, Täby, Sweden) and stained for CD11b (BD, Franklin Lakes, USA) or CD14-APC (BD, Franklin Lakes, USA) in blocking buffer for 2 h. After washing with PBS, the PBMCs were incubated with a secondary goat anti-mouse Alexa Fluor 568 antibody (Invitrogen, Waltham, MA, USA) diluted in PBS for 40 min. After washing, the cells were stained with Hoechst 33,342 (Thermo Fisher Scientific) and mounted with Prolong Diamond Antifade mount (Thermo Fisher Scientific). Imaging was performed with an SP8 confocal microscope (Leica, Wetzlar, Germany), with LAS X version 3.5.5 software (Leica). Brightness and contrast were adjusted using Adobe Photoshop version 24.6.0 (Adobe, San Jose, CA, USA).

### Histopathological examination

Skin biopsy samples collected on day 3 post-infection were fixed with 4% phosphate-buffered formaldehyde, embedded in paraffin, and sectioned with a microtome. Tissue sections were thereafter stained with hematoxylin and eosin. All the slides were coded and assessed under a microscope in a blinded manner by two observers (T.J. and M.M.).

### Fibrin(ogen) immunohistochemical staining

Tissues were deparaffinized and hydrated with water. An antigen retrieval procedure was performed using the 2100-Retriever device (PickCell Laboratories, Lelystad, the Netherlands) and DIVA Decloaker (Biocare Medical, Pacheco, CA, USA). Slides were blocked with 3% bovine serum albumin (BSA) and then incubated overnight at 4°C with horseradish peroxidase-coupled goat antibodies against mouse fibrin(ogen) (Accurate Chemical & Scientific Corporation, Westbury, NY, USA). Slides that were treated with 1% BSA instead of antibodies served as negative controls. Color was developed with 3,3′-diaminobenzidine (DAB) and counterstained with Harris’s hematoxylin solution (Sigma-Aldrich) (53). Slides were evaluated under a microscope in a blinded manner by T.J. and M.M.

### Statistical analysis

All statistical analyses were performed using GraphPad Prism version 9.4.0 software for Macintosh (GraphPad Software, La Jolla, CA, USA). Statistical significance was assessed using the Mann-Whitney *U*-test, Fischer’s exact test, and Mantel-Cox log-rank test, as appropriate. The results are reported as the mean ± standard error of the mean (SEM) unless indicated otherwise. A *P*-value < 0.05 was considered statistically significant. The number of repeats for each experiment is described in the associated figure legends.

## Data Availability

The authors declare that the main data supporting the findings of this study are available within the article and its supplemental material. Additional supporting data are available from the corresponding author upon request.

## SUPPLEMENTAL MATERIAL

Supplemental material mbio.00840-26-s0001.pdf Supplemental figures and table.

ASM does not own the copyrights to Supplemental Material that may be linked to, or accessed through, an article. The authors have granted ASM a non-exclusive, world-wide license to publish the Supplemental Material files. Please contact the corresponding author directly for reuse.
