# Supplementary material for: Staphylococcal lipoproteins and peptidoglycans synergize to drive skin abscess formation
Source: mBio. 2026 May 15;17(6):e00840-26. doi: 10.1128/mbio.00840-26 (PMC13251356; doi:10.1128/mbio.00840-26)
Supplement: Supplemental material — Supplemental figures and table. [file mbio.00840-26-s0001.pdf]

Staphylococcal lipoproteins and peptidoglycan synergize to drive skin abscess formation

Majd Mohammad[1], Zhicheng Hu[1,2], Julia M. Scheffler[1], Mulugeta Nega[3], Arif Luqman[3,4], Malgorzata Krzyzowska[1,8], Martina Sundqvist[1], Pradeep Kumar Kopparapu[1], Rille Pullerits[1,5], Abukar Ali[1], Minh-Thu Nguyen[6], Friedrich Götz[3], Tao Jin[1,7*]

[1]Department of Rheumatology and Inflammation Research, Institute of Medicine, The Sahlgrenska Academy, University of Gothenburg, Gothenburg, Sweden

[2]Department of Microbiology and Immunology, The Affiliated Hospital of Guizhou Medical University, Guiyang, China

[3]Department of Microbial Genetics, University of Tübingen, Tübingen, Germany

[4]Biology Department, Institut Teknologi Sepuluh Nopember, Indonesia

[5]Department of Clinical Immunology and Transfusion Medicine, Sahlgrenska University Hospital, Gothenburg, Sweden

[6]Section of Medical and Geographical Infectiology, Institute of Medical Microbiology, University Hospital of Münster, Münster, Germany

[7]Department of Rheumatology, Sahlgrenska University Hospital, Gothenburg, Sweden

[8]Department of Pharmaceutical Chemistry and Biomaterials, Faculty of Pharmacy, Medical University of Warsaw, Poland

* Corresponding author

Supplementary Fig. 1. Staphylococcal lipoproteins and peptidoglycan synergically induce skin lesions.

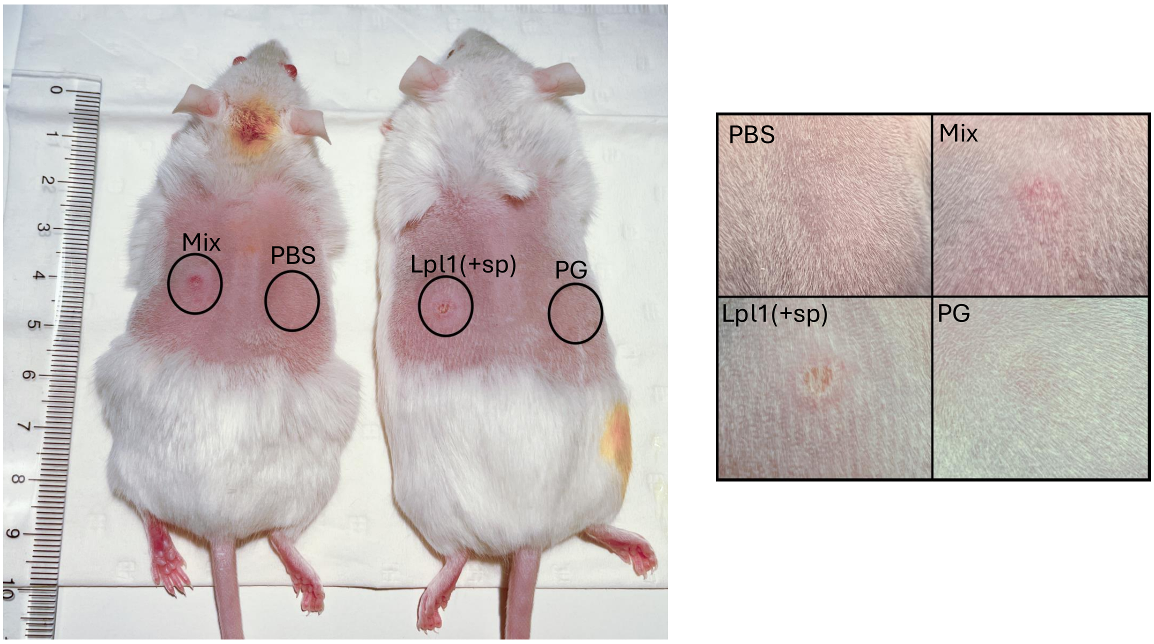

Representative images of skin lesions on NMRI mice on Day 3 following subcutaneous skin injection (20 µl/site) of PBS, Lpl1(+sp) (2.5 µg/site), peptidoglycan polymer undigested (PG) (10 µg/site), or co-injection of Lpl1(+sp) (2.5 µg/site) and PG (10 µg/site).

Supplementary Fig. 2. Levels of cytokines in skin tissues obtained from mice that were injected with purified components of *S. aureus*.

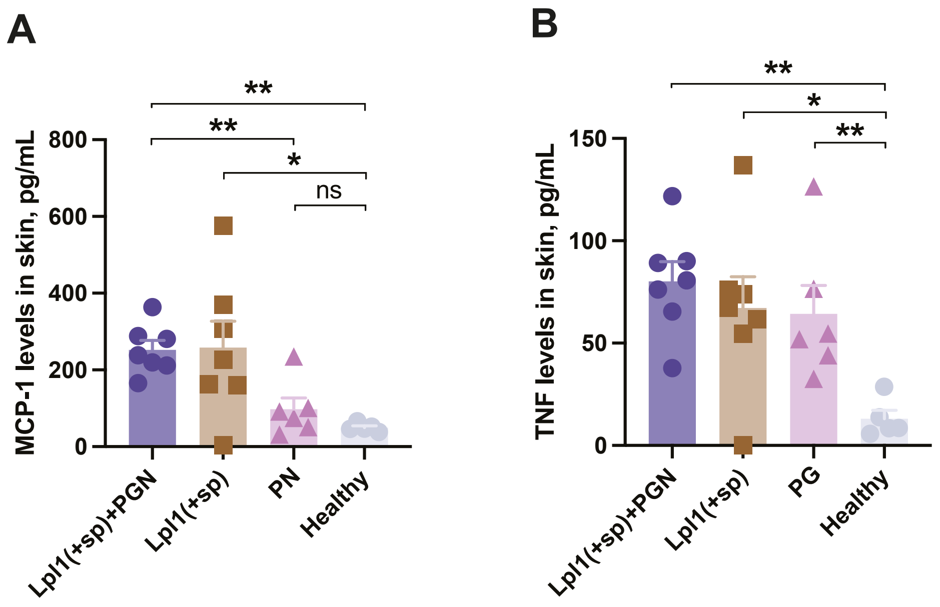

The levels of (**A**) monocyte chemoattractant protein 1 (MCP-1) and (**B**) tumor necrosis factor alpha (TNFα) in the supernatants of skin biopsy homogenates obtained from healthy (n = 5) NMRI mice or on Day 3 after subcutaneous skin injection of 20 µl of Lpl1(+sp) (2.5 µg/site, n = 7), PG polymer undigested (PG) (10 µg/site, n = 6), or co-injection of Lpl1(+sp) (2.5 µg/site) and PG polymer undigested (10 µg/site, n = 7). The data were pooled from two independent experiments. Statistical evaluations were performed using the Mann-Whitney *U*-test, with data presented in a scatterplot with the mean ± standard error of the mean (A-B). *$P < 0.05$; **$P < 0.01$; ns = not significant.

Supplementary Fig. 3. Growth curves of Newman parent strain (WT) and its mutant strains including Δ*lgt,* Δ*oat, and* double-mutant Δ*oatA*Δ*lgt* strain.

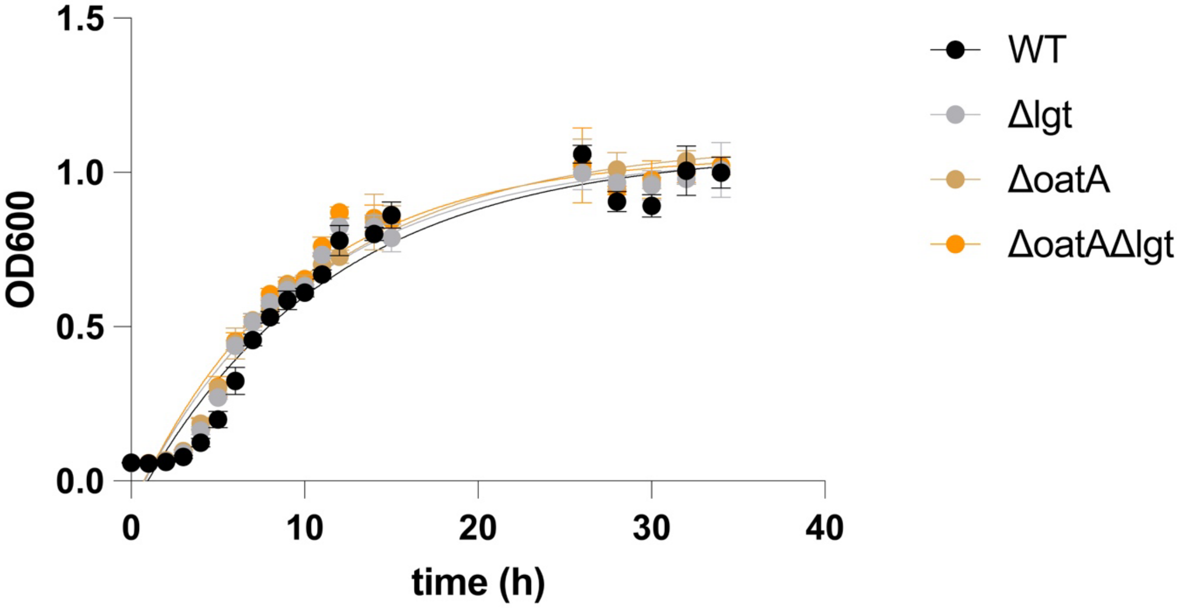

The strains were first streaked on horse blood agar plates and grown overnight to obtain single colonies. Three single colonies from each strain were collected and each incubated in 10 mL of TSB broth at 37°C with shaking at 180 rpm. At the indicated time points, 100 µL of bacterial suspension was collected, and the optical density at 600 nm (OD600) was measured using a spectrophotometer to determine bacterial growth over time for up to 36 hours.

Supplementary Fig. 4. Differences in skin lesion severity and bacterial clearance between the parental strain and the Δ*oatA*Δ*lgt* double-mutant strain are independent of fibrinogen depletion.

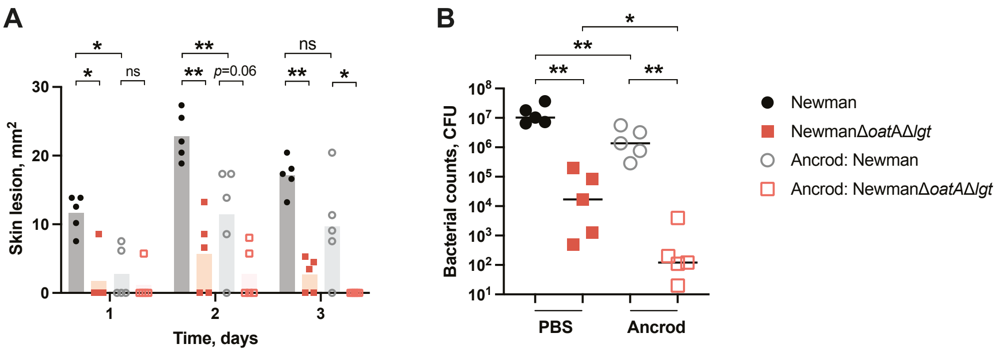

The skin lesion size (mm^2) at up to 3 days post-injection (**A**) and the bacterial counts in the supernatants of skin biopsy homogenates (**B**) on Day 3 after subcutaneous (s.c.) skin injection with 50 µl of *S. aureus* Newman parental strain or NewmanΔ*oatA*Δ*lgt* double-mutant strain (4 × 10^6 colony-forming units/site) into NMRI mice depleted of fibrinogen using Ancrod or treated with phosphate-buffered saline (PBS) as control (n = 5/group). Statistical evaluations were performed using the Mann-Whitney *U*-test, with data presented in a scatterplot with the mean (A), or presented as a scatterplot with line indicating the median value (B). $*P < 0.05$; $**P < 0.01$; ns = not significant.

Supplementary Fig. 5. HPLC analyses of the PGpolymer and PGmonomer preparations. A. Distinct peaks corresponding to the PG polymer and monomer fractions are spectrophotometrically detected at 205 nm. B. The high purity of the PG monomer product is verified by HPLC analysis.

A

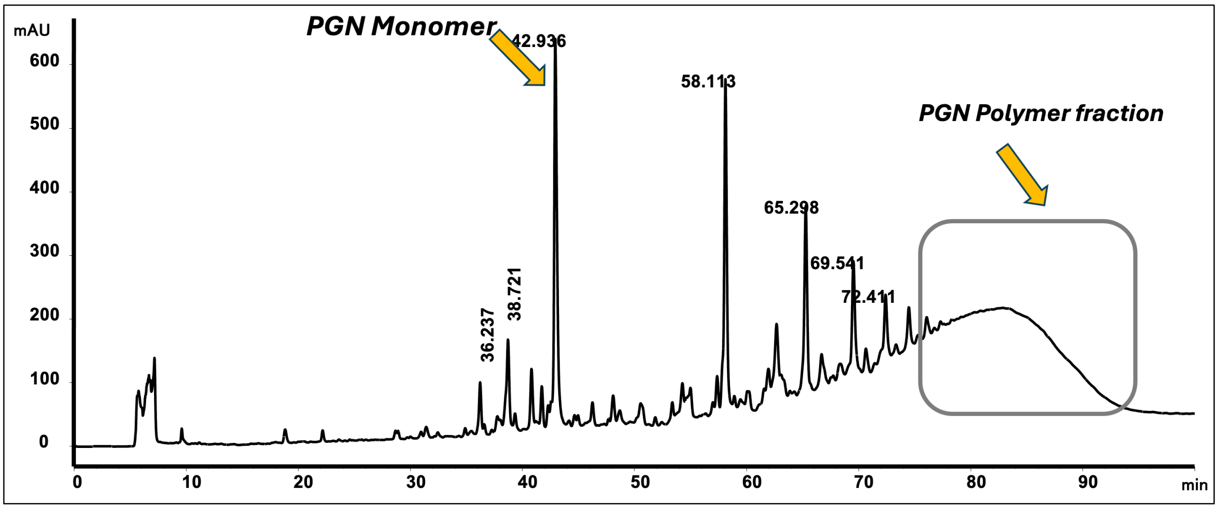

B

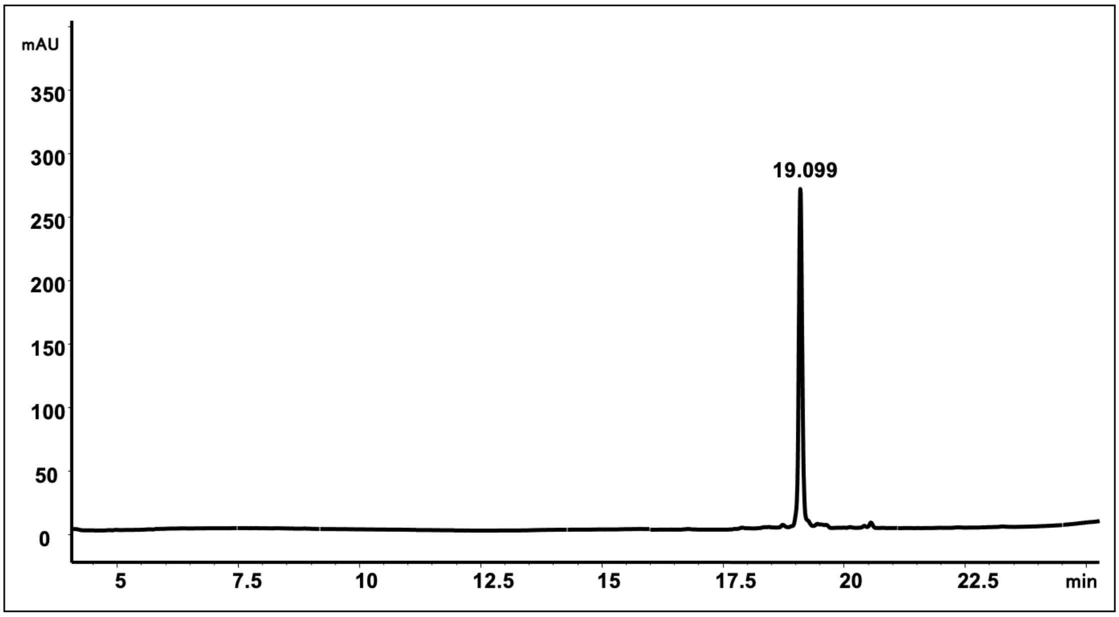

Supplementary Table 1. Primers used to generate the *S. aureus* Δ*lgt* and Δ*oatA* mutants. Approximately 1-kb upstream (Up) and downstream (Down) regions of *lgt* and *oatA* were amplified using the listed primers.

Primer	Sequence 5′–3′
Up pBASE lgt KO F	CAC TCA TCG CAG TGC AGC GGT TTT GAT ATG ATA AGA AGA GAT GTA AG
Up pBASE lgt KO R	ACT ACT TCA CAA TAC CCA TTC AAC CTA CTC
Down pBASE lgt KO F	AAT GGG TAT TGT GAA GTA GTG ATA GTT TGA G
Down pBASE lgt KO R	GCC CGG GTA CCG AGC TCC GGT ACT ACA CGA TGA TCT TGA AC
Up pBASE oatA KO F	CAC TCA TCG CAG TGC AGC GGA TGG CTA TAA ACA TAA TTG AAT ATA ATA G
Up pBASE oatA KO R	ATT ATT TCT TTG TAT CCA TGT TAA TAA ACG C
Down pBASE oatA KO F	CAT GGA TAC AAA GAA ATA ATT TGA TGC ACT AAA CTT TTG
Down pBASE oatA KO R	GCC CGG GTA CCG AGC TCC GGA CGT AAG CAA CAT GAC CG

F, Forward primer; R, Reverse primer.
